# Microwave Properties of Metacomposites Containing Carbon Fibres and Ferromagnetic Microwires

**DOI:** 10.34133/2019/3239879

**Published:** 2019-04-11

**Authors:** Yang Luo, Diana Estevez, Fabrizio Scarpa, Larissa Panina, Huan Wang, Faxiang Qin, Hua-Xin Peng

**Affiliations:** ^1^Institute for Composites Science Innovation (InCSI), School of Materials Science and Engineering, Zhejiang University, 38 Zheda Rd., Hangzhou 310027, China; ^2^Bristol Composites Institute (ACCIS), University of Bristol, Queens Building, University Walk, Bristol BS8 1TR, UK; ^3^National University of Science and Technology, MISiS, Moscow 119991, Russia; ^4^Institute of Physics, Mathematics & IT, Immanuel Kant Baltic Federal University, A. Nevskogo 14, 236041 Kaliningrad, Russia

## Abstract

The microwave properties of composites containing Fe-based ferromagnetic microwires and carbon fibres have been investigated as part of a campaign to bring added functionalities into structural composites. A transmission window observed in 1-6 GHz demonstrates double-negative (DNG),* i.e.*, metamaterial characteristics in the composites containing short-cut carbon fibres and a parallel array of microwires; the metamaterial characteristic is due to the ferromagnetic resonance and a plasmonic behaviour, as short carbon fibres are proved to ameliorate DNG properties through enhancing the impedance of the composites. In parallel, magnetically tunable metamaterial features are realised in composites containing continuous carbon fibres and microwires, which can be switched on/off via rotating the electrical excitation direction. Such structural composites integrated with metamaterial features (termed as metacomposites) are potentially useful for active cloaking applications among others.

## 1. Introduction

“Metamaterial” is a currently widely used term referring to certain materials/systems/devices that exhibit “unusual properties that are not observed in conventional natural materials,” such as the electromagnetically double negative (DNG) feature, which is attractive to a number of ingenious applications ranging from invisibility cloaking to high-resolution imaging [1–3]. DNG property is defined by a negative dielectric permittivity and a negative magnetic permeability that are simultaneously available in the frequency regime of interest, and with an easy design of the topology [4]. However, issues such as narrow DNG bandwidth, poor tunabilities, and high manufacturing costs of metamaterials have limited their industrial exploitation [5]. In this context, as part of a campaign to bring added functionalities to structural composites, we have designed and manufactured “metacomposites” where metamaterial features are integrated with a structural glass-fibre reinforced polymer (GFRP) composite via embedding ferromagnetic microwires [6, 7]. As elucidated in a recent work [6], these metacomposites differ from conventional metamaterials on three basic aspects: (i) they are materials rather than structures, or systems of structures; (ii) they can be produced by using conventional composites manufacturing techniques, instead of micro-/nanofabrication techniques to make complicated building blocks; and (iii) their ultimate properties are dependent not only on the geometric parameters of the architecture, but also on the intrinsic characteristics of the fillers. Thus far, Fe-based ferromagnetic microwires have been selected as “functional fillers,”* i.e.*, “building blocks” in these metacomposites due to their tunable ferromagnetic resonance and tailorable geometrical characteristics [8]. Many interesting features in the microwire metacomposite category have been obtained and understood,* e.g.*, DNG [6, 9], tunable negative permittivity [10, 11], and magnetic/stress tunable properties [12, 13]. These metacomposites are also relatively inexpensive to manufacture, because the embedded microwires can be fixed within the microstructure of the glass/epoxy composites with a classical curing process, as opposed to expensive nanotechnologies that are needed to fabricate conventional metamaterials [14]. Furthermore, an extremely low concentration of microwires (<0.035 wt.%) is normally sufficient to enable DNG properties; this low weight fraction favours the miniaturisation of devices due to the microwires' high sensing capability towards external stimuli such as magnetic field, stresses, and temperature [15–17].

Compared to GFRP composites, carbon fibre reinforced polymer (CFRP) composites are more widely used in aerospace as load-bearing components due to their light weight, high structural strength, and excellent anticorrosion abilities [18, 19]. Embedding microwires into composites containing carbon fibres (CFs) to develop single negative (SNG) or DNG properties is of enormous practical significance in the integration of,* e.g.*, zero-loss structural health monitoring, microwave cloaking, and perfect absorbing functions into existing CFRP components. The outstanding mechanical properties of the microwires can guarantee the overall structural integrity of the developed metacomposites. However, due to electrically reflective nature of CFs and hence the difficulty to extract useful EM parameters from the CFRP composites, there exists two challenges: (i) the concentration of carbon fibres should be carefully designed [20]; (ii) the orientation of carbon fibres needs to be considered which causes the metamaterial features highly anisotropic to external excitation directions.

In the present work, as a step further to our previous focus on GFRP metacomposites [6], we have made our first attempt to design and manufacture metacomposites containing Fe-based microwires and carbon fibres. Important DNG features are verified via the observation of a transmission window in the 1-6 GHz range in the composite containing short-cut CFs. This composite also features a significantly smaller reflection compared to GFRP metacomposites. Metacomposites containing continuous CFs and microwires exhibit a highly anisotropic metamaterial behaviour where magnetically tunable permittivity and permeability are present with the microwires arranged along the electrical field.

## 2. Materials and Methods

**Figure 1:**
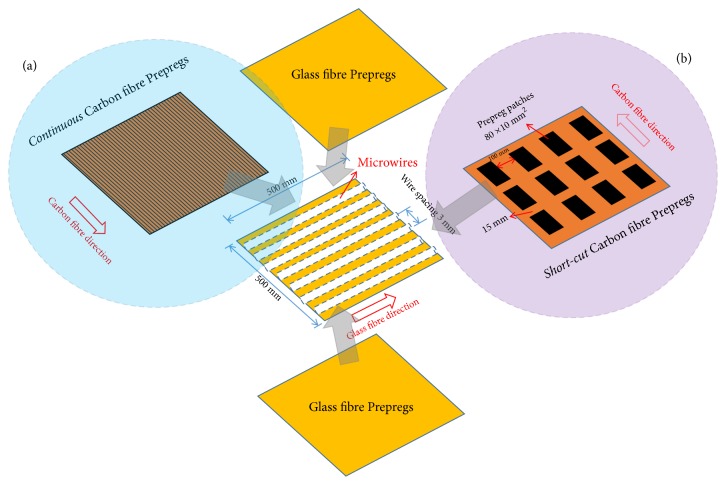
Schematic illustration of metacomposites containing 3 mm spaced parallel Fe-based microwires and (a) continuous and (b) short-cut carbon fibres.

**Figure 2:**
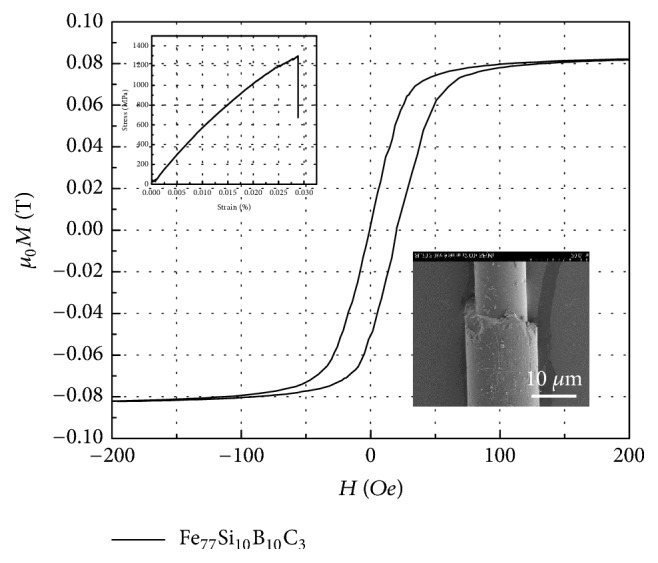
Magnetic hysteresis (M-H) profiles of Fe_77_Si_10_B_10_C_3_microwires. The insets are their tensile properties and SEM images.

Amorphous glass-coated Fe_77_Si_10_B_10_C_3_microwires with the total diameter of 20*μ*m and a Pyrex glass coating of 1.7 *μ*m were fabricated by a modified Taylor technique [21–23]. Processing parameters such as wheel rotating speed and cooling water distance were carefully selected to promise a near-perfect cylinder geometry with decent roundness and surface (inset of Figure 2) [24]. The microwires were embedded into an aerospace-graded carbon fibre prepreg of 500×500 mm^2^(IM8557, Hexcel) in a parallel manner with the spacing of 3mm. One should note that the aligning direction of the microwires should be perpendicular to the direction of the carbon fibres to minimise reflection loss, as indicated in Figure 1(a). In this stage, a microwire-CF prepreg layer was formed (continuous version). In another geometry (short-cut version), the carbon fibre prepregs were cut and arranged in smaller patches (80×10 mm^2^) as shown in Figure 1(b) and then were included with the microwire array. Preliminary trials indicated that the simple exposure of CFs on the surface of the wire-composites was not the way forward, since the microwave signals were fully blocked and prevented from further interacting with the microwires. Instead, the above microwire-CF prepreg layer should be included as the middle prepreg layer and sandwiched between additional glass-fibre prepreg panels that are microwave-transparent during the laying-up process. Two additional glass fibre prepregs of same size (500×500 mm^2^) were therefore used to host the microwire-CFs prepreg layer (Figure 1). The whole metacomposite was then cured in autoclave for curing (details can be found in [13]). Microwave measurements were carried out by using a free-space technique in the 0.9-17 GHz with an external dc magnetic bias swept between 0 and 3000 A/m. The* S*-parameters were extracted during the measurement and then calculated into permittivity and permeability using the Nicolson-Ross-Weir method via a modified built-in computing programme [25].

## 3. Results and Discussion

Figure 2 describes the magnetic, mechanical, and structural properties of the Fe-based microwires used for the metacomposites. A square-shaped magnetic hysteresis (M-H) loop is observed with a magnetisation saturation of 810 Gs and a small coercivity of 25 Oe; those values suggest the presence of soft magnetic properties. The rectangular loop is due to the longitudinal magnetic anisotropy of the Fe-based microwires that induces a large Barkhausen effect in the magnetisation process [8]. The linear tensile stress-strain curve indicates a typical brittle fracture behaviour at small strains due to the amorphous nature of the microwires. The tensile strength however reaches 1280 MPa, which is beneficial to maintain the mechanical performance of the microwire metacomposites; this attribute is much desirable and advantageous as it is often a concern that the added functionality with introduced fillers will inevitably result in a compromise of otherwise excellent structural property [26].

**Figure 3:**
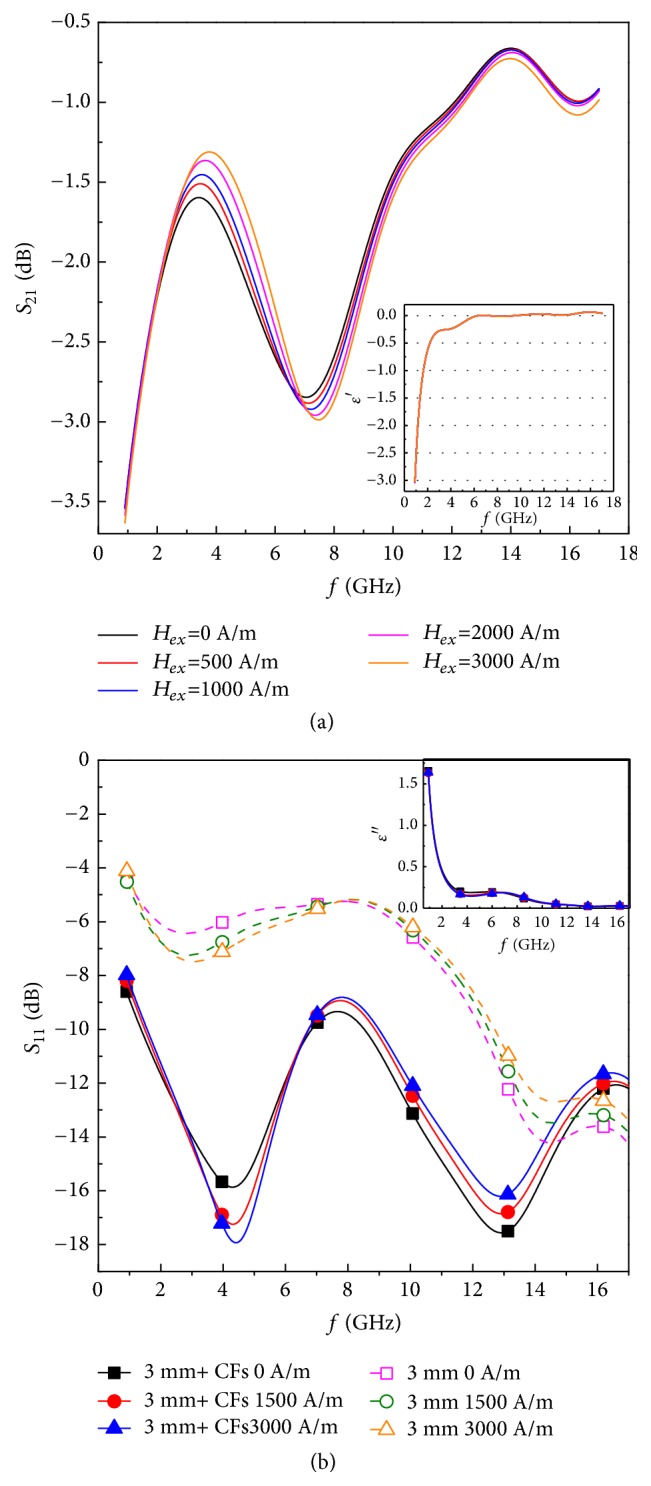
Frequency dependencies of (a) transmission* S*_21_ and (b) reflection* S*_11_ coefficients of the short-cut CFRP metacomposites in the frequency range of 0.7-17 GHz under external magnetic bias up to 3000 A/m. The insets of (a) and (b) are the real and imaginary part of permittivity of the metacomposites, respectively.

Figure 3 shows the transmittance and permittivity of the metacomposite containing short-cut CFs and microwires within the 0.7-17 GHz range. Transmission windows are clearly observed in the frequency band of 1-6 GHz (Figure 3(a)). Previous works reveal that the enhancement of the transmission can be derived from double positive or DNG indices [27–29]. However, all negatives in the real part permittivity spectrum observed in the 1-6 GHz band are a clear indication of the DNG properties of our metacomposite (inset of Figure 3(a)). The negative transmission phase velocity also gives evidence to the existence of this DNG feature. The negative permittivity is determined by the plasma frequency via a classic Drude model, which in the metamaterial sense can be simplified to

(1) $$\begin{array}{c} f_{p}^{2}=\frac{c^{2}}{2\pi b^{2}\ln\left(b/a\right)} \end{array}$$

where* a* is the radius of wires,* b* is the spacing, and c is the speed of light [30]. Taking* a*= 8.3*μ*m (radius of metallic core) and* b*= 3mm we obtain* f*_p_= 16.3 GHz. Therefore, the permittivity should be negative below 6 GHz as evidenced by the permittivity spectra (inset of Figure 3(a)). Meanwhile, permeability values should be negative between the ferromagnetic resonance (FMR) frequency and the ferromagnetic anti-resonance (FMAR). The FMR frequency of the microwires is read as

(2) ωFMR=μ0γH0+MsH0,

(3) H0=Hex+Ha

where *γ* is the gyromagnetic ratio, *μ*_0_ is the vacuum permeability,* Ms* is magnetisation saturation,* H*_a_ is the magnetic anisotropy, and* H*_ex_ is external magnetic field, respectively [27]. From Figure 2 one can obtain* H*_a_=30 Oe, and by substituting* H*_ex_=0 Oe the* f*_FMR_ is calculated as 0.9 GHz. This value coincides with the frequency range of the transmission window. Hence, the obtained DNG values can be attributed to the synergistic action between topology and magnetic properties of the microwires. More importantly, the transmittance level of the metacomposite containing short CFs and wires is enhanced compared with the metacomposite containing parallel wires alone;* e.g.*, the highest transmittance increases from -5.6 dB (52.5%) [6] to -1.6 dB (83.2%) at zero field. The transverse conductivity in the unidirectional CFRP composite is well known to be rather poor due to the electrically insulating matrix resin [20]. Noting that **E** is placed perpendicularly to the CFs, as a consequence, the magnitude of the permittivity of the wire-composites should be decreased with the insertion of CFs, leading to an increase of the impedance, and an increase of the transmittance of the metacomposites containing carbon fibres. The reduced permittivity is also verified by the presence of an imaginary part of the measured permittivity (inset of Figure 3) with magnitude below 1.8, suggesting a very weak dielectric response.

Of particular note is that the metacomposite containing wires and carbon fibres exhibits significantly lower reflection coefficients in the frequency range where the transmission windows are realised, as opposed to the metacomposite containing 3 mm spaced wires (Figure 3(b), see also [6]). One can conclude that the low transverse conductivity of the CFs patches (in the direction perpendicular to CFs) favours the extraction of left-handed signals in their wire-composite. This feature further guarantees the presence of significant interactions among the transmitted waves in the composites containing short CFs and wires, which benefits the DNG response by enhanced transmitting signals.

**Figure 4:**
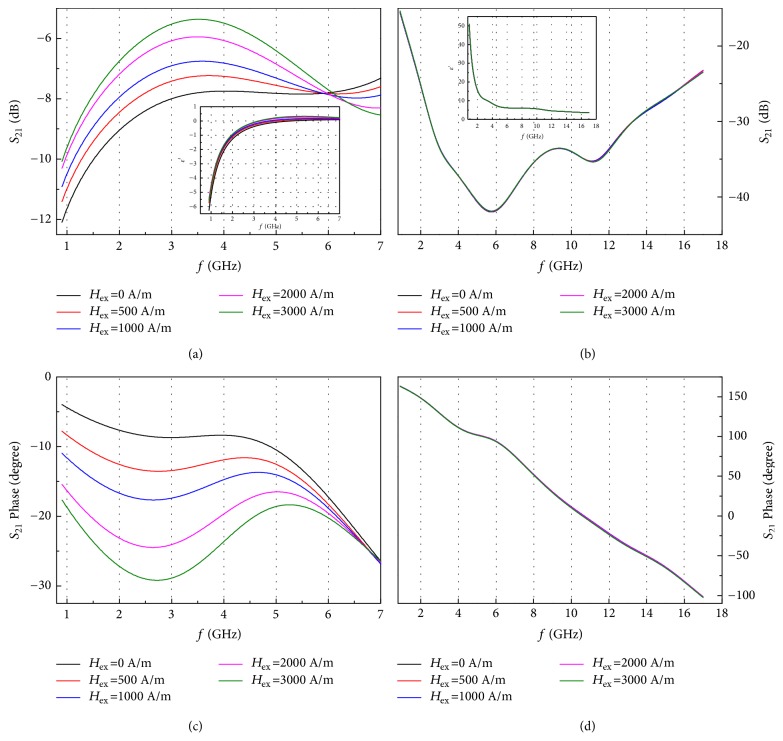
Frequency plots of transmission spectra of continuous carbon fibres contained hybrid metacomposites with **E** placed (a) along and (b) perpendicularly to microwires. Frequency plots of transmission phase of the same composite in (a)/(b) with **E** (c) along and (d) perpendicular to microwires. The insets in (a) and (b) are the frequency dependences of their *ε*′ when **E** is along and perpendicular to microwires, respectively.

Despite the presence of a strong DNG feature, the microwave behaviour of metacomposite containing short-cut CFs and wires is hardly tunable with the presence of magnetic field (Figure 3(a)). Further, a prepreg layer containing continuous CFs and wires (Figure 1(a)) is introduced to improve the impedance match of the wire-composites and hence the tunability.  Figure 4 describes the transmission and phase of the metacomposite containing continuous CFs and microwires in the 0.7-17 GHz band with magnetic fields up to 3000 A/m. When the electrical component of the microwaves is oriented along the direction of the microwires, a series of transmission windows are obtained under different magnetic fields (Figure 4(a)). The change of phase velocity reversing from negative to positive together with the negative permittivity (inset of Figures 4(a) and 4(b)) suggests that the DNG properties of such metacomposite still persist. There are two reasons for this: first, the dielectric epoxy in the prepregs prevents the movement of free charges amongst the carbon fibres. The parallel microwire array can be therefore regarded as a dielectric plasma if **E** is oriented along direction of the wires, therefore favouring the onset of negative permittivity values [31]. The second reason is the presence of an insulating layer of glass on the wires, which can prevent again the movement of free charges from the microwires to the conductive polymer matrix. The electrons are therefore trapped within the metallic cores, making the microwires perfect electric conductors. On the other hand, the transmittance level is slightly decreased compared with the metacomposite containing 3mm spaced microwires,* e.g.*, from -5.6 (52.5%) to -7.8 dB (40.7%) at zero field [6], and this is due to the increase of the overall conductivity when a significant amount of conductive carbon fibres is integrated in the composite.

It is also worth noticing that the magnitudes of the observed transmittance windows are linearly dependent on the increase of external magnetic fields;* i.e.*, the waves can be quantitatively controlled to pass the microwire metacomposite by applying an external magnetic bias (Figure 4(a)). The maximum amplitude variation under different fields reaches 2.4 dB at 3.5 GHz, and this represents 78.5 % of the total incident microwave signals. Quite interestingly, the embedding of the microwires into the composites with continuous CFs still preserves the DNG properties if the microwires are overlapped with the carbon fibres in an orthogonal manner.

As per Kittel's equation [32], the windows should be tuned towards higher frequencies as the external fields increase. Nevertheless, hard contacts between microwires and reinforcing carbon fibres would create stress-concentration points that may lead to breakage of the surface glass, or even the damage or failure of the wires. The magnetic anisotropy would be also different from the value retrieved from the M-H results (Figure 2), resulting in less tunable state when external magnetic fields are applied.

Neither evident field-tunable nor DNG features are observed in the metacomposite containing continuous CFs and microwires when the electric field is oriented along the direction of the CFs (Figures 4(b) and 4(c)). The transmittance of the composites in this case is rather low;* e.g.*, the lowest transmission is obtained as -41.9 dB (0.8 %) as seen in Figure 4(b). In this scenario, double positive indices are present, with most electromagnetic signals reflected. The transmission phase and permittivity results also suggest the presence of a double positive medium for the composite (Figure 4(d) and inset of Figure 4(b)) because neither ferromagnetic resonance nor dielectric plasma can be excited to realise negative permittivity and permeability values. The comparison of the results shown in Figures 4(a) and 4(c) suggests the presence of an interesting phenomenon: the switch between left-handedness and right-handedness indicated by a conversion of the double-positive to double-negative permittivity values, which is identified by rotating the direction of **E**. This direction-dependent microwave characteristic of the metacomposites containing continuous CFs and microwires can activate or suppress the metamaterial characteristics by rotating the electrical excitation by 90 degrees. To the best of the authors' knowledge, this significant microwave direction dependency is identified for the first time in metacomposites with a parallel wires configuration, and it is reminiscent of a similar feature obtained in orthogonal microwire metacomposites [7]. The orthogonal microwire architecture still requires the presence of a parallel array of microwires in the fibre reinforcing direction due to magnetic interactions among wires that can contribute to the generation of negative permeability and hence of the DNG properties [7]. Obviously, such a presence of those wires is a burden design as additional amount of wires is required. Nonetheless, in the present work we manage to attain a very anisotropic metamaterial behaviour by building up a simple parallel stacking geometry of microwires and by reducing the total amount of microwires. In a brief summary, two propagation modes are observed in the metacomposite containing continuous CFs and microwires,* i.e.*, highly transmitting mode with DNG indices, and a highly reflective mode with double positive indices. From the application point of view, anisotropic metamaterial functionalities open up opportunities to develop active microwire cloaks. A composite material with the intrinsic characteristics shown in this work would be able to change its status from cloaking/exposure by adapting its orientation versus the electrical polarisation, with no detriment to its mechanical performance.

## 4. Conclusions

The paper provides evidence of the generation of electromagnetic metamaterial properties in composites containing carbon fibres and arrays of unidirectional Fe-based microwires. The metacomposite integrated with short-cut carbon fibres and wires features a transmission window within the 1-6 GHz band due to the plasmonic behaviour of array of wires and their FMR. Such DNG property is also observed in the metacomposites containing continuous CFs with the condition that **E** is along microwires, which otherwise will transform to show a double-positive phenomenon induced by the disappearance of the FMR effect and the plasma excitation. Our metacomposites containing carbon fibres and microwires provide a proof-of-concept that shows it is possible to use ferromagnetic microwires-impregnated composites for,* e.g.*, active microwave cloaking applications.
